# The nature and structure of maternal parenting practices and infant behaviors in U.S. national and international samples

**DOI:** 10.3389/frcha.2023.1124037

**Published:** 2023-05-26

**Authors:** Marc H. Bornstein, Diane L. Putnick, Gianluca Esposito, Rebecca M. Pearson

**Affiliations:** ^1^ Intramural Program, Eunice Kennedy Shriver National Institute of Child Health and Human Development, National Institutes of Health, Bethesda, MD, United States; ^2^ UNICEF, New York City, NY, United States; ^3^ Institute for Fiscal Studies, London, United Kingdom; ^4^ University of Trento, Trento, Italy; ^5^ Manchester Metropolitan University, Manchester, United Kingdom

**Keywords:** culture, parenting, mother-infant interaction, infancy, modeling

## Abstract

**Methods:**

Twenty maternal parenting practices and 15 behaviors of their 5½-month-old infants in a U.S. national sample (*N* = 360) and 9 international samples (*N* = 653) were microcoded from videorecords of naturalistic interactions at home and aggregated into domains. Altogether, the samples were recruited from Argentina, Belgium, Brazil, France, Israel, Italy, Japan, Kenya, as well as the United States.

**Background and Rationale:**

A previous test of three competing models of the nature and structure of the maternal parenting practices supported a hybrid 2 factor/6 domain model as superior to a 1-factor dimensional model and a multi-factor style model: Maternal parenting practices are structured into nurture, physical, social, didactic, material, and language domains undergirded by dyadic and extradyadic factors. Infant behaviors were organized into physical, social, exploration, nondistress vocalization, and distress communication domains. The current study sought to examine links connecting these previously identified maternal domains and factors with infant behavior domains using structural equation models.

**Results:**

Mothers' dyadic factor is associated with infant social behaviors with mother; and mothers' extradyadic factor and encouragement of infant physical development are associated with infant exploration of their immediate physical environment and physical development. Infant distress communication (and less nondistress vocalization) is associated with more maternal nurturing.

**Discussion:**

Mothers' parenting practices in the middle of the first year of infant life are commonly structured and adapted to specific needs and developmental tasks of infants. Evaluations of mother-infant interactions with national and international samples permit a wide yet judicious analysis of common vs. specific models of mother-infant relationships.

## Introduction

### Parenting and infancy at the beginning of life

As approximately 370,000 babies are born each day, hundreds of thousands of new mothers around the globe experience the joys but also assume the daunting responsibilities of new parenthood (1). Parents are fundamentally invested in their infants' survival and subsistence as well as their socialization and education. Moreover, infants actively shape their own development, and so parents and infants influence one another bidirectionally and transactionally (2). A compelling tension pervades the dynamic of this foundational human relationship. On the one hand, specific parent-infant dyads have idiosyncratic needs and goals, which are shaped by unique and individual bioecocultural forces. On the other hand, human parents and infants everywhere display many of the same biopsychosocial needs and must succeed at many of the same caregiving and developmental tasks. Indeed, at the end of the day parents everywhere want physical health, mental achievement, social adjustment, and economic security for their children—however those goals may be locally instantiated. At different levels of analysis, then, parenting and infancy likely vary in specific ways faithful to individual and social differences but also share commonalities that transcend those individual and social differences. The main aims of the complementary pair of studies presented here were to assess the nature and structure of maternal parenting practices and infant behaviors as well as commonalities and specificities of mother-infant relationships in U.S. national and 9-country international samples from Argentina, Belgium, Brazil, France, Israel, Italy, Japan, Kenya, as well as the United States.

We report the videorecording, microcoding, analysis, and emergent organization of relationships between maternal parenting practices and infant behaviors as enacted during naturalistic interactions at home in 360 primiparous U.S. European American and 653 primiparous mothers from 9 different countries with their 5½-month-old infants. Undertaking an evaluation of parent-infant relationships in the context of a broad national and international research design permits a comprehensive yet judicious determination of the nature and structure of mother-infant relationships as well as their commonalities and specificities. These studies also contribute information about parenting and infancy in several still relatively underresearched populations and compare it to parenting infants in populations that have been more comprehensively studied.

### Maternal parenting practices, infant behaviors, and their interaction: three aims

#### Maternal parenting practices

Parents endow their offspring with a genetic makeup, and they supply and share experiences with their children and so co-construct the environments in which their children are reared. Indeed, before children are old enough to enter formal social learning situations, like school, or even informal ones, like peer groups, virtually all of children's experiences stem directly from interactions they have within the family. In this light, parents' cognitions and practices directly influence infant development and prepare infants for wider social interactions (3).

A previous report attempted to gain greater purchase on the nature and structure of mothers’ parenting in international settings. Individual parenting practices in primiparous mothers with their 5½-month-old infants in one U.S. European American sample and samples from 9 different nations were microcoded from videorecords of naturalistic home-based mother-infant interactions, and three possible models of caregiving were compared (4). Out of the varied repertoire of individual caregiving activities, six parenting domains that meet fundamental developmental tasks of infants were derived. The six domains included, in precis, *nurturant* caregiving that meets the biological, physical, and health requirements of infants through such practices as feeding, thermoregulation, grooming, and clothing. *Physical* caregiving promotes infants' gross and fine psychomotor balance and movement. *Social* caregiving includes visual, verbal, affective, and physical activities that engage infants in interpersonal exchanges, express affection, and involve social play as well as regulate affect and emotions. *Didactic* caregiving draws infants' attention to properties, objects, and events in the environment as well as labels and describes, demonstrates and stimulates. *Material* caregiving provisions and organizes infants' home and local environments, including the number, variety, and composition of inanimate objects available to the infant, level of ambient stimulation, limits on physical freedom, and overall physical components of infants' experiences. *Language* caregiving is a major channel through which mothers maintain contact, interpret infant cues, respond to infants, introduce experiences, and express affection. Taken as a totality, this constellation of parenting domains constitutes a varied and demanding set of caregiving tasks that together encompass virtually all of parents' important activities with their infants. They are likely universal, even if their qualitative instantiation (e.g., meaning) or quantitative emphases (e.g., frequency or duration) vary across cultures. Reciprocally, human infants universally are reared in, influenced by, and adapt to social and physical environments that are characterized by the domains of this parenting taxonomy.

Further analysis determined that these six domains converged on two conceptually independent, developmentally significant caregiving factors (4). Akin to the dimensional school of caregiving, parenting infants was characterized by a *Dyadic* factor which included the nurture, physical, social, didactic, and language domains that optimally engage infants in warm, nurturing, supportive interpersonal exchanges, and by an *Extradyadic* factor which included the didactic, material, and (negatively loading) nurture domains that incorporate properties, objects, and events in the natural and designed environments and stimulate infants to engage and understand their surround. Many investigators have previously theorized and operationally distinguished between dyadic and extradyadic parenting as separate and significant whether they are called animate vs. inanimate, affective vs. informational, or social vs. didactic (e.g., Field, 1981; Goldfield, 1987; Sherrod, 1981; Stern, 1985). This hybrid 2 factor/6 domain model of parenting held for a U.S. sample as well as a sample including 9 international countries and appears to be robust as it obtained for mothers with daughters and sons and over and above variation in maternal age, education, and personality.

#### Infant behaviors

To complement that study of mothering, here we incorporate infant behaviors. Fifteen individual infant behaviors that are key expressions of early performance and development were also microcoded from videorecords of the same naturalistic mother-infant interactions. The 15 individual infant behaviors are organized into five infant behavior domains—physical, social, exploration, nondistress vocalization, and distress communication. These five domains account for most or all of young infants' principal adaptive competencies. That is, infants are growing, discovering, communicating, relating … and complaining beings … who exercise effectance however they can given the constraints of babyhood (5). We did not expect any factorial organization among the five infant domains because previous work has shown that infant behavioral domains share minimal amounts of their common variance (6).

#### Mother-Infant interactions and three aims

Infancy is the phase of the life cycle when adult parenting is thought to exert extremely salient influences: Not only is the sheer amount of interaction between parent and offspring greatest in infancy, but young human infants are totally dependent on parents and especially susceptible and responsive to their experiences (7). Indeed, opportunities for enhanced parental influences and prolonged childhood learning are thought to constitute evolutionary reasons for the extended duration of human infancy (8). In the two studies presented here, activities of primiparous mothers and their 5½-month infants were analyzed from naturalistic interactions at home. The principal mother-infant interaction issue investigated concerns the degree to which maternal parenting practices and infant behaviors correspond (i.e., correlate) with one another. Testing this issue in the context of a single US sample and international sample advances our understanding of specificities and commonalities of everyday family life.

The two studies had three main aims. One aim was to explore relations of the two maternal parenting practice factors and six domains to the five infant behavior domains in a U.S. sample. The U.S. sample was chosen as the target because it was the largest sample available and the originial protocol was developed in the United States. In the test of the first aim, out of 40 possible associations between mother and infant domains/factors, we hypothesized 7 paths. We expected paths from mother dyadic focus to infant physical, social, and nondistress vocalization, from mother extradyadic focus to infant physical, exploration, and nondistress vocalization, and from infant distress communication to mother-infant dyadic focus. These associations were hypothesized because mother dyadic focus includes behaviors expected to promote infants' physical development, social interactions, and responsive vocalizations. Furthermore, mother extradyadic practices focus the infant on the surrounding environment, which likewise promotes physical and exploratory behaviors as well as nondistress vocalizations. Although associations between mother and infant activities are inherently dyadic, most hypothesized relations were from mothers to infants, recognizing the prominent role mothers play in guiding interactions with their young infants. The exception was infant distress communication. The most effective means the infant commands to communicate is crying, which elicits powerful and universal responses in mothers (9), and justifies hypothesizing the direction of effect from infant to mother.

To date, the vast majority of the extant literature in developmental science, and consequently our understanding of human parenting and infancy, derives from studies conducted in so-called WEIRD (Western, educated, industrialized, rich, and democratic) nations (10). Where there are exceptions, precious little standardization has been brought to bear on comparative examinations of even the most basic parenting and developmental constructs, structures, functions, or processes (11, 12). The second aim of the present studies was to explore whether the model derived from the U.S. sample applied to a diverse international sample.

The sex of an infant is often a principal interest in developmental science (13). The third aim of the present studies was to assess whether the model of mother-infant interaction was moderated by the sex of their infants.

### Biology and experience: commonality and specificity in parenting and infancy

Some signal characteristics of parenting appear to be “wired” into the biological makeup of the human species (14), and adults already know (or think they know) some about parenting by the time they first become parents. For example, parents routinely speak to their infants even though they know that babies cannot understand language, and they even speak to babies in a special speech register (“infant-directed speech”) that modifies adult-directed speech in prosodic, simplicity, redundancy, lexical, and content features (15, 16). Infant-directed speech is intuitive, nonconscious, and cross-culturally common (17, 18); even deaf mothers modify their sign language to babies in much the same ways (19). Likewise, the same brain regions in mothers of different cultures are excited by infant cries, and mothers in different cultures respond in behaviorally similar ways to infant cries (20, 21). At the same time, human beings acquire parenting cognitions and practices through specific experiences: Generational, social, and media images of parenting, children, and family life play equally significant roles in helping people form their parenting cognitions and guide their parenting practices (22, 23). For example, parents from different cultures differ in the ages they expect children to reach different milestones or acquire various competencies, and they differ in their opinions about the significance of specific competencies for their children's success in social adjustment (24). The origins of variation in maternal cognitions and practices are multivariate and complex and include biological processes and psychological attributes of parents, actual or perceived characteristics of infants, and contextual influences, including social situations, socioeconomic status, and ethnicity and culture (3).

### These studies

The two studies presented here aimed to uncover more about mother-infant interaction in the period of the dyad's initial mutual accommodation in the middle of the first year of the infant's life by analyzing multiple activity domains of mothers and infants in different national samples. As key and possibly long-lasting characteristics of individuals arise in early life, mother-infant interaction is thought to be at least one important source of human development. The overall strategy was initially to use U.S. parent-infant data [capitalizing on its large *N* and which in (4) revealed a 2 factor/6 domain structure to parenting] to determine the best-fitting model of the structure of mother-infant relationships and then to evaluate whether the best-fitting structure also fit a larger international sample.

## Two studies: general methods, procedures, and analytic plan

The nature and structure of mother-infant interactions were assessed to address three aims in two companion studies: one single-nation and one international. Are maternal parenting practice domains and factors related to infant behavior domains in identifiably patterned ways in a U.S. sample? Is the pattern of maternal parenting-infant behavior relationships similar for an international sample? Are mother-infant relationships moderated by infant sex in a U.S. and an international sample? Approaches to these aims were based on extensive and detailed standardized observations and systematic analyses of naturalistic parenting practices of new primiparous mothers and the behaviors of their young infants.

### Participants in studies 1 and 2

A total of 923 primiparous mothers and their healthy 5½-month-old infants from Argentina, Belgium, Brazil, France, Israel, Italy, Japan, Kenya, and the United States participated in the two studies. Details about the samples appear in descriptions of Study 1 and Study 2 participants below. Justification for the focus on mothers appears in the Supplementary Material. Mothers were recruited from hospital or published birth notifications, patient lists of medical groups, newspaper advertisements, and targeted mailings. Mothers who expressed a willingness to participate in home-based naturalistic observations with their infants and who, with their infants, satisfied the following developmental and sociodemographic criteria were included in Studies 1 and 2 on first-come-first-recruited bases. Mothers were at least 16 years of age at their child's birth and were living in intact families; infants were firstborn only children, born at term, weighed more than 1,500 g at birth, healthy, and 5½ months of age on average at the time of the observation. Approximately equal numbers of female and male infants were enrolled into each country sample. In addition to the mother-infant dyads that met these inclusion criteria and participated, 5 dyads were excluded from Study 2: 2 dyads from France and 2 from Japan were excluded because the durations of their videorecords that could be coded totaled less than 42 min (of an expected hour), and 1 infant from Argentina was excluded because the baby slept more than 5 min during the first 50 min of recording.

Infants were studied in the middle of the first year because of the intentionality and flexibility in behavioral organization which most normally developing infants demonstrate at this time. No longer *fetus ex utero*, by the middle of the first year the infant's scope of perception has broadened to the dyad and beyond, infants actively participate in turn-taking exchanges, and they show readiness and ability to explore the world outside the dyad, looking, touching, and mouthing objects with increasingly extensive and efficient exploration. For their part, caregivers encourage infants of this age to attend to properties, objects, and events in the environment, and they provision the infant's environment with toys, books, and other objects that vary in quality and quantity and fill the infant's environment with sounds of different kinds, notably their own language.

### General procedures applicable to studies 1 and 2

#### Procedures

Approximately 1 h of naturalistic interaction of mothers with their infants was videorecorded, microcoded, and analyzed for each dyad. Meta-analyses have indicated that maternal practices are most stable for observations lasting 30 min to 1 h compared to shorter or longer observations (25, 26); briefer observations can be unstable, and lengthier observations are likely to include highly varied activities and contexts (27). In these studies, attempts were made to remain faithful to the principle of ecological validity by focusing on naturalistic interactions between mothers and infants in their own home setting; that is, the goal was to observe spontaneous activities of the two under the most natural and unobtrusive conditions possible. Studying dyads at home presumably maximized their comfort and increased the validity of the observations. All observations were also conducted in a standardized way to render the data comparable across diverse samples. Briefly, mothers were asked to behave in their usual manner; videographers were always young women native to the country; mothers were instructed to disregard the videographer's presence insofar as possible; beside the videographer, only mother and infant were present in the home; and observations took place at times of the day when infants were awake and alert. Recording commenced only after a conventional period of acclimation to the presence of the videographer and the camera [as recommended in (28, 29)].

#### Maternal parenting practice and infant behavior codes

The development of censuses of maternal parenting practices and infant behaviors involved extensive observations, collaborative discussions, and intensive analyses. First, narrative observational accounts of maternal parenting practices and infant behaviors were made in the field. Field testing and refinement were then conducted. In this way, initial, unstructured descriptive data were shaped into structured observations and, ultimately, quantitative data. Subsequently, formal operational definitions of maternal parenting practices and infant behaviors were developed to facilitate coder accuracy and consistency (see Supplementary Tables S1, S2). These definitions represent discrimination rules for coding target activities, and they met three main criteria: (1) the definitions were objective and referred to directly observable practices and behaviors; (2) the definitions were clear, unambiguous, and easily understood so that trained coders could accurately use them; and (3) the definitions required little or no inference. Going forward, the phrases “maternal parenting practices” and “infant behaviors” are used; however, the abbreviated generic term “activity” is used to capture the two as appropriate.

#### Maternal parenting practices

Eighteen individual parenting practices and two context indicators constitute primary parenting tasks and performance competencies of mothers of young infants. Together, they aggregate into the six parenting practice domains listed in Supplementary Table S1 Column 1. Each practice and context constituent indicator is operationally defined in Supplementary Table S1 Column 2.

#### Infant behaviors

Fifteen individual infant behaviors constitute key developmental and performance competencies that are critical to ontogenetic adaptation in young infants. Together, they aggregate into five infant behavior domains listed in Supplementary Table S2 Column 1. Each behavior constituent indicator is operationally defined in Supplementary Table S2 Column 2.

#### Coding

The 18 maternal parenting practices and two context indicators and the 15 infant behaviors were coded from the videorecords *via* computer entry. Coding of sets of practices and behaviors took place on multiple individual passes through the videorecords and, depending on the practice or behavior, was continuous and comprehensive or time-sampled or consisted of counts and ratings. Continuous and comprehensive coding was implemented for single or mutually exclusive and exhaustive sets of conceptually related activities. In mutually exclusive coding, only one activity can be coded for each unit of observation; in exhaustive coding, one activity must be coded for each unit of observation. Only activity initiations needed be recorded because in the software used the onset (start) of a new activity automatically signaled the offset of the preceding activity (30). A set of objective parameters was programmed into the software such that the minimum duration of an activity was set to .30 s, and an interruption of an activity for less than 1 s did not constitute a new instance of the activity. Continuous and comprehensive coding is rigorous and powerful, yielding unbiased estimates of the frequency and duration of activities on the basis of their occurrence in the uninterrupted, natural time flow (31). Mutually exclusive and exhaustive coding enjoys numerous conceptual and statistical advantages [see (30–33)]. In time sampling, whether or not an activity occurred during a fixed time interval was recorded (34, 35). Finally, some activities were counted or rated. With coded videorecords in hand, detailed analyses of maternal parenting practices and infant behaviors were undertaken.

Narrowly defined and specific activities are generally easier for coders to learn and to apply, require less interpretation and inference, and can later be aggregated for summary data analyses (as done here in the conversion of activities into domains). Concrete and basic mother and infant activities were studied, and they have been observed previously in samples from each nation [albeit at varying levels (20, 21)]. Microcoding categorizes overt activities at high temporal resolution and captures detailed information from observational data. Microcoding and microanalysis are traditional in parenting and infancy research (36–40).

#### Coders

A small group of trained coders was employed, and coders addressed and resolved issues that arose on account of different national samples and coder bias. First, as data from different national samples were to be coded, the needs for multiple trained reliable coders with multiple ethnic heritages and checks on measurement techniques arose. To address these problems, coding focused on recording the onsets (and so the frequency and duration) of activities rather than global ratings. Moreover, all coders were required to become reliable with a set of standardized reference codings. Second, coders may miss information—the human visual and auditory senses can be insensitive or unreliable in detecting certain activities—and coders can suffer from information overload. When a large number of target activities must be coded in a short period of time, a coder may have difficulty detecting or recording all of them. Sometimes coders harbor or develop (correct or incorrect) hypotheses about the nature and purpose of an investigation, how participants should behave, or what constitute “appropriate” data. Thus, coders may make systematic errors and hold biases based on their information-processing limitations and expectations.

To address issues of coder bias and to maintain the accuracy of quantitative measures, coders and coding adhered to standardized procedures (41): (1) Only trained and experienced but naïve coders were recruited, and stringent training criteria were employed. Only coders who possessed the ability to sustain attention, who had a propensity for detail and precision and a commitment to scientific detachment, and who were analytically minded were recruited and trained. (2) Prior to actual coding, coders were trained to criterion performance accuracy and consistency on a series of standard videorecords which had varied and representative samples of the target mother and infant activities. Coders learned to code accurately, and the pressure of time was eliminated. (3) Videorecords were randomly assigned to coders. (4) Coders were cautioned about the potential negative effects of bias, and they remained naïve to the specific scientific aims of these studies. (5) To the degree possible, precise low-inference operational definitions of activities were used. Coders learned the operational definitions and scoring procedures of the observation system as presented in a formal training manual. (6) Coding drift was corrected by means of regular reliability checks with experienced coders and the standardized codings. Coders knew that their codings would be routinely checked for reliability but did not know which specific codings would be used. If “drift” away from coding accuracy occurred, re-training sessions were conducted (42–44).

#### Coding reliability

Different statistical metrics of coder reliability were employed. For all continuously coded activities, *Kappa* (*κ*) (45, 46) was used. *Kappa* is observed agreement beyond that expected by chance as a proportion of possible agreement beyond that expected by chance. *Kappa* was based on agreement in each 5-s interval for parenting practices and infant behaviors. *Kappa*s were always evaluated relative to the prevalence index, the bias index, and the maximum attainable *κ* (47, 48). For time-sampled activities, the *Intraclass Correlation* (*ICC*) (49–51) in two-way random effects models was used. After coders achieved initial reliability, at least every tenth videorecord that they coded was independently coded by second coders. Between 11% and 37% of each national sample (depending on the nation and domain) was coded independently by pairs of coders to monitor intercoder reliabilities. Supplementary Table S3A gives intercoder reliabilities of mothers' parenting practice domains by country; averages for the 6 maternal parenting practice domains across the 9 countries were: Nurture *κ* = .89, Physical *ICC* = .72, Social *κ* = .67, Didactic *κ* = .73, Material *ICC* = .85, and Language *κ* = .70. Supplementary Table S3B gives intercoder reliabilities of infant behavior domains by country; averages for the 5 infant behavior domains across the 9 countries were: Physical *ICC* = .92, Social *κ* = .59, Exploration *κ* = .70, ICC = .88, Nondistress vocalization *κ* = .68, and Distress communication *κ* = .69.

#### Data

Coding maternal parenting practices and infant behaviors generated multiple measures which are termed interim variables of frequency and duration, proportion, and variety, density, and consistency (Supplementary Tables S1, S2 Column 3). Continuously coded activities generated both frequency and duration interim variables. Frequency is the number of discrete times a mother or infant engaged in an activity or, more precisely, the number of times the activity was initiated during the observation. Duration is the total time that a mother or infant engaged in the activity. Practices in the nurture domain yielded only duration interim variables. Time-sampled activities yielded a single interim variable, the proportion of observation units in which the activity occurred. Context indicators of quantity and quality of objects yielded counts and ratings of three interim variables: variety, density, and consistency.

Supplementary Tables S1, S2 Column 4 specify how interim variables were aggregated into final indicator variables for each activity domain. Indicator variables for continuously coded domains are mean standard scores (*z*-scores where *M* = 0, *SD* = 1) of the frequencies and durations of the interim variables over the first 50 min of the observation. Indicator variables which were derived from time-sampled practices are also mean standard scores. Except for mother nurture, domain scores were computed as the simple mean of the indicator variables for the domain, the indicator variables being equally scaled interim variables (mean standard scores, proportions). Because the indicator variables for mother nurture are duration scores of unequal means and standard deviations, the domain score was computed as the mean of the standard scores of the indicator variables. Supplementary Tables S1, S2 Column 5 specify how indicator variables were aggregated to form mean standard scores of final domain variables.

Videorecords were first microcoded at level of the indicators (31, 52). To eliminate unnecessary complexity and realize clearer pictures of mother-infant interaction, dependent indicator variables were aggregated at domain levels, and analyses reported here are restricted to domains. Restricting analyses to domains takes full advantage of the multiple benefits of dimension reduction (53). Supplementary Tables S1, S2 Column 1 list the maternal parenting practice and infant behavior domain names. Column 2 lists individual activities that were coded and their operational definitions. Column 3 lists the interim variables derived from coding those activities. Column 4 lists the final indicator variables derived from the interim variables. Column 5 lists the mean standard scores of final variables that constitute a domain and on which analyses were conducted.

The concept of an activity domain is akin to an index and distinct from the concepts of a factor or latent variable. The latter refer to unobserved constructs that manifest in, and are inferred from, several theoretically and empirically related indicator variables. The domains as used here consist of conceptually related practices or behaviors that may, or may not, be empirically or statistically related (54, 55). Justification for inclusion in a domain is conceptual coherence (*qua* an index) and not necessarily empirical relatedness (*qua* a scale). Preliminary analyses and the analytic plan appear in the Supplementary Material.

## Study 1: mother-infant interactions in a United States sample

### Study 1: the U.S. Sample

The U.S. sample consisted of 360 European American primiparous mothers and their 5½-month-old infants, 162 mother-daughter and 198 mother-son dyads. Participants were recruited from the greater Washington, DC metropolitan area, including suburbs of Maryland, Virginia, and rural West Virginia. Sociodemographic information for participating U.S. mothers and infants appears in Table 1. Mothers averaged 29.4 years of age (*SD* = 6.2, range = 16.3–43.1). Mothers' average educational level as measured on the Hollingshead Index with (56) 7-point education scale was 5.6 (*SD* = 1.3). Families were middle-class on average [SES; (56), Four Factor Index of Social Status: *M* = 50.1, *SD* = 12.9; see also (57)]. Infants averaged 163.4 days of age (*SD* = 6.1, range = 141 to 195) when observed. An ethnically homogenous European American community sample was recruited, first because a majority of the population of the United States identifies as European American (58), and second as an initial step toward understanding the nature and structure of mother-infant interactions in advance of embarking on the more complex follow-up Study 2 and analysis with nationally diverse samples (59, 60).

**Table 1:** Demographic characteristics of the U.S. sample.

	Total U.S. Sample (*N* = 360)
Mothers’ age (years)	29.4 (6.2)
Mothers’ education^a^	5.6 (1.3)
Infants’ age (days)	163.4 (6.1)
Infants’ gender (% females)	45%
Infants’ birth weight (grams)	3,493.3 (508.1)
Family SES^b^	50.1 (12.9)

*M*(SD). Reproduced in part from Bornstein et al. (4).

^a^ Hollingshead (56) 7-point scale.

^b^ Hollingshead (56).

### Study 1: U.S. sample results

#### Descriptive statistics

Supplementary Tables S4A,B present the means and standard deviations for each maternal parenting practice and infant behavior domain score for the U.S. sample.

#### Mother-Infant interaction model

**Figure 1:**
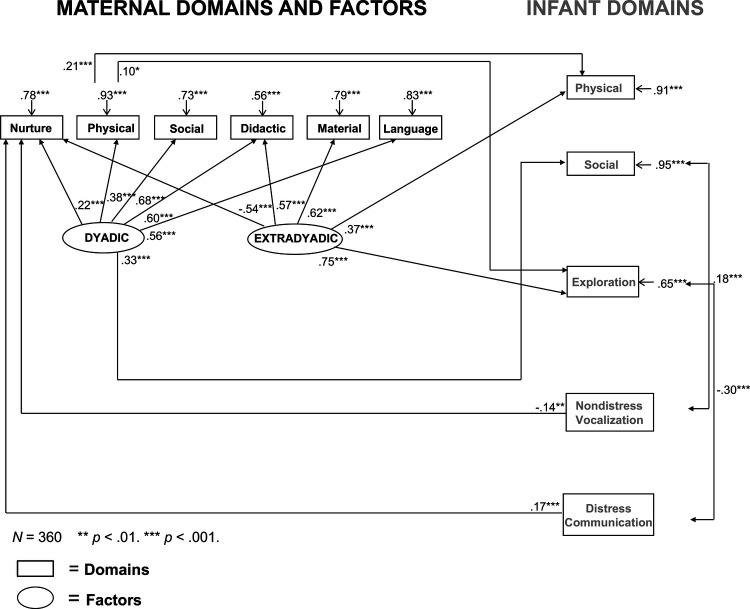
From videorecords of naturalistic mother-infant interactions at home in a U.S. sample (*N* = 360), 20 maternal parenting practices and 15 behaviors of their 5½-month-old infants were microcoded. The 20 maternal parenting practices aggregated into 6 domains and subsequently into 2 factors, and the 15 infant behaviors aggregated into 5 domains. The standardized solution for the final mother-infant interaction model is shown in figure. Numbers associated with single-headed arrows from maternal factors to maternal domains and from maternal domains or factors to infant domains are standardized path coefficients; numbers associated with double-headed arrows are standardized covariance estimates; and numbers asociated with single-headed arrows and maternal or infant domains are error or disturbance terms, the amount of variance not accounted for by paths in the model.

Figure 1 presents the standardized solution to the structural portion of the final, sequentially constructed model, S-B *χ*^2^(38) = 79.31, *p *< .001, Robust CFI = .93, RMSEA = .06, 90% CI = [.04, .07]. Modifications of the hypothesized model included (1) dropping associations of maternal dyadic and extradyadic focus with infant nondistress vocalization; (2) replacing the path between maternal dyadic focus and infant physical development with a path from mother encouragement of physical behavior to infant physical development; (3) adding a path from mother encouragement of physical behavior to infant exploration; (4) replacing the path between infant distress communication and maternal dyadic focus with a path from infant distress communication and mother nurture; and (5) adding a (negative) path from infant nondistress vocalization to maternal nurture. In addition, two pairs of variances/unique variances for infant behaviors were allowed to covary: social and nondistress vocalization, and exploration and distress communication (negative). Infant social and nondistress vocalization were allowed to correlate because babies may babble while engaging in face-to-face interaction with their mothers. Exploration and distress communication were allowed to correlate negatively because distressed babies are unlikely to engage with objects in their environment. The model reproduced observed correlations with an average absolute standardized error of .03. All parameters estimated in the model were significant at the .05 level or stronger. Supplementary Table S5 presents the correlation matrix, variances, and standardized residuals for the final model.

The maternal dyadic factor related positively to the infant social domain: Mothers who engaged in more dyadic focus had infants who engaged in more social exchanges with their mothers. The maternal extradyadic factor and unique variance of the mother physical domain related positively to the infant physical and exploration domains: Mothers who encouraged their infants' physical development more and mothers who engaged in more extradyadic interactions had infants who exhibited higher levels of motor development and explored their environments more. Two infant behaviors contributed to the time mothers spent in nurturing them: Mothers whose infants vocalized nondistress less and communicated distress more nurtured their infants more.

#### Infant sex

To test whether the final model fit equally well for mother-daughter and mother-son dyads, a series of nested multisample models that sequentially introduced constraints on the measurement model path coefficients and covariances was constructed (61). All factor loadings, factor variances, and unique variances were constrained to be equal. A preliminary test in which no parameter estimates were constrained to be equal fit the data, *χ*^2^(76) = 116.49, *p *< .01, CFI = .94, RMSEA = .06, 90% CI = (.03, .07), suggesting that more restrictive models were appropriate. In testing the series of nested multisample models, a consistent pattern emerged in which two path coefficients (infant distress communication to mother nurture and mother physical to infant exploration) and one unique variance (mother nurture) differed for mothers of female and mothers of male infants; there were no differences in any other parameter estimates tested. In the most rigorous test in which all parameters except these three coefficients were constrained to be equal, an adequate fit was achieved, *χ*^2^(101) = 149.98, *p *< .01, CFI = .92, RMSEA = .05, 90% CI = [.03, .07]. The difference in *χ*^2^ between this model and the model that imposed no invariance constraints was not significant, *χ*^2^(25) = 33.49, *p *= .12, suggesting that, with the exceptions of the two path coefficients and one unique variance, imposing invariance across measurement model (as described above), path coefficients, and covariances had no deleterious effects on model fit. The two path coefficients which showed a sex difference were the paths from infant distress communication to mother nurture (standardized coefficient = .30, *p *< .001, for mothers of female infants and .06, *p *= .34, for mothers of male infants) and from mother physical to infant exploration (standardized coefficient = .22, *p *< .001, for mothers of female infants and .02, *p *= .75, for mothers of male infants): Distress communication of female infants was associated with their mothers' nurturing, and mothers who more often encouraged their female infants' physical development had female infants who engaged in more exploring. No parallel relations existed in mother-son dyads. The unique variances associated with mother nurture (standardized unique variances = .44 for mothers of female infants and .68 for mothers of male infants, *p*s < .001) differed statistically between mothers of female infants and mothers of male infants; however, both were significant.

### Study 1: discussion

European American first-time mothers' parenting practices with their young infants have a hybrid bifactorial (dyadic and extradyadic) structure that is expressed in six parenting domains [nurture, physical, social, didactic, material, and language (4)]. Infant behaviors aggregate into five behavioral domains (physical, social, exploration, nondistress vocalization, and distress communication). In terms of their associations, European American first-time mothers who engage in more dyadic interactions have infants who engage in more social exchanges with their mothers; and mothers who engage in more extradyadic interactions and mothers who encourage their infants' physical development more have infants who exhibit higher levels of motor development and explore their environments more. Finally, mothers whose infants communicate distress more (and vocalize nondistress less) nurture their infants more. The overall structure of European American first-time mothers' parenting infants largely obtains for mothers with daughters and mothers with sons. The General Discussion sets these results in broader empirical and theoretical contexts.

## Study 2: mother-infant interactions in an international sample

According to a survey of 10 international journals concerned with psychological aspects of infancy, 92% of articles published between 2002 and 2012 were based on WEIRD populations (62). In brief, too little is still known about parenting, infancy, and mother-infant interaction across a broad swath of the world's nations. In consequence, Study 2 was designed to evaluate the nature and structure of mother-infant interactions in 9 diverse national samples. To the extent that research in developmental science is dominated by WEIRD samples, it is challenging to distinguish universal processes from those specific to WEIRD societies. Some universals in parenting, infancy, and mother-infant relationships likely exist, as parenting infants in different places likely draws on the same human neural, mental, and emotional machinery, just as infants likely elicit similar responses from parents that may be requisite for their wholesome development. However, human activities are known to vary (sometimes quite dramatically) across populations in different places, and mother-infant interactions constitute one such prominent activity.

### Study 2: the international sample

Altogether 653 primiparous mothers and their healthy 5½-month-old infants were recruited into the international sample from Buenos Aires and Córdoba Province, Argentina (*n* = 139), Ghent and Antwerp, Belgium (*n* = 117), Rio de Janeiro, Brazil (*n* = 40), Paris, France (*n* = 59), Haifa, Israel (*n* = 31), Padua and Ruoti, Italy (*n* = 100), Tokyo, Japan (*n* = 47), and the Kamba tribe, Kenya (*n* = 30); additionally, data from a subsample (*n* = 90) of mother-infant dyads from the United States from Study 1 were used in Study 2. That subsample, stratified by maternal age and education and selected randomly, did not differ from the U.S. Study 1 sample in the means and variances of three key sociodemographic characteristics (mothers' age and education and family SES). As the same factor structure and comparable model fit indices were obtained with and without the U.S. mothers in the total international sample, reported results are based on the total international sample (including U.S. mothers).

Table 2 presents sociodemographic information for all participants in each country. Mothers averaged 27.7 years of age (*SD* = 4.6, range = 16.2–44.0). Because differences exist between countries in the duration, quality, and content of schooling, bicultural researchers adjusted mothers' years of schooling in each nation so that all education scales were equivalent to the Hollingshead U.S. scale. Mothers' average educational level as measured relative to the Hollingshead Index (56) 7-point education scale was 4.6 (*SD* = 1.3). Families in the 9 international samples were middle-class on average as measured by the Hollingshead (*M* = 43.2, *SD* = 12.6). Infants averaged 161.1 days of age (*SD* = 8.0, range = 131–198).

**Table 2:** Demographic characteristics of the international samples.

	Mothers’ Age		Mothers’ Education^a^		Infants’ Age^b^		Infants’ Sex	Infants’ Birth Weight^c^		Family SES^d^	
	*M*	*SD*	*M*	*SD*	*M*	*SD*	% females	*M*	*SD*	*M*	*SD*
Argentina (*n* = 139)	25.3	4.9	3.9	1.4	165.1	8.3	46.8	3,343.5	462.3	34.5	14.6
Belgium (*n* = 117)	29.3	3.6	5.2	1.1	157.1	9.7	47.0	3,405.9	453.2	47.6	11.0
Brazil (*n* = 40)	25.8	5.9	3.9	1.9	156.7	5.6	52.5	3,349.7	379.2	36.4	13.9
France (*n* = 59)	30.8	4.7	5.4	1.3	166.0	10.9	44.1	3,252.5	366.5	53.4	10.7
Israel (*n* = 31)	28.0	3.5	5.4	0.7	166.3	4.7	51.6	3,346.2	433.6	50.9	6.7
Italy (*n* = 100)	27.4	4.5	3.5	1.5	154.9	5.1	50.0	3,247.5	388.4	33.4	13.6
Japan (*n* = 47)	29.0	2.9	5.6	0.9	162.0	9.1	51.1	3,039.8	364.9	52.8	11.0
Kenya (*n* = 30)	21.7	3.4	2.5	1.7	159.1	9.1	50.0	2,913.7	558.9	–	–
U.S. (*n* = 90)	29.6	6.0	5.6	1.3	164.1	6.3	43.3	3,539.9	537.3	50.3	12.9
Total (*N* = 653)	27.7	4.6	4.6	1.3	161.1	8.0	47.6	3,317.7	446.1	43.2	12.6

Reproduced in part from Bornstein et al. (4).

^a^ Hollingshead (56) 7-point scale.

^b^ Days.

^c^ Grams.

^d^ Hollingshead (56).

### Study 2: international sample results

#### Descriptive statistics

Supplementary Tables S6A,B present the means and standard deviations for each maternal parenting practice and infant behavior domain score for all participants and by each country separately.

#### Mother-Infant interaction model

**Figure 2:**
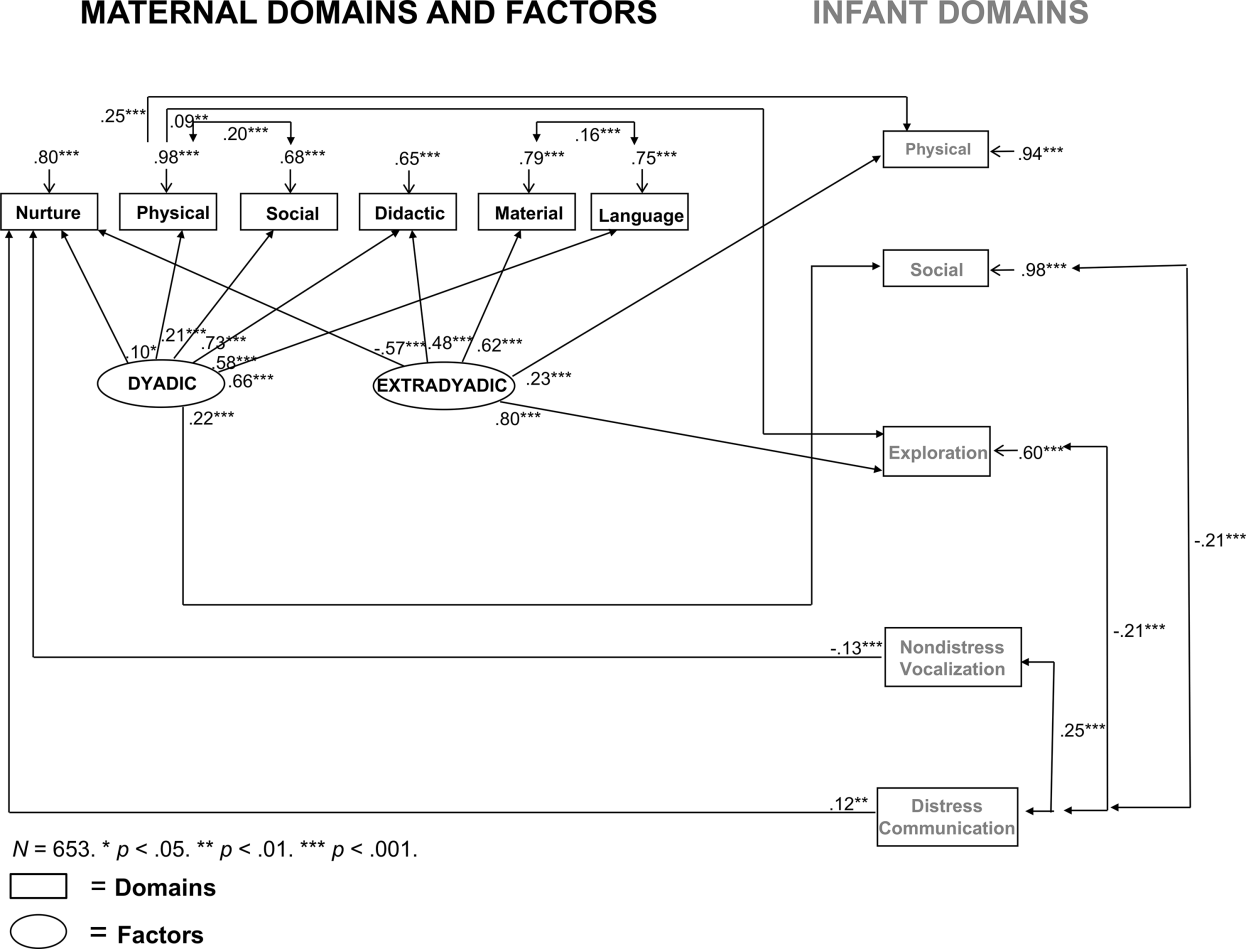
From videorecords of naturalistic mother-infant interactions at home in an international sample, aggregated from Argentina, Belgium, Brazil, France, Israel, Italy, Japan, Kenya, and the United States (*N* = 653), 20 maternal parenting practices and 15 behaviors of their 5½-month-old infants were microcoded. The 20 maternal parenting practices aggregated into 6 domains and subsequently into 2 factors, and the 15 infant behaviors aggregated into 5 domains. The standardized solution for the final mother-infant interaction model is shown in figure. Numbers associated with single-headed arrows from maternal factors to maternal domains and from maternal domains or factors to infant domains are standardized path coefficients; numbers associated with double-headed arrows are standardized covariance estimates; and numbers asociated with single-headed arrows and maternal or infant domains are error or disturbance terms, the amount of variance not accounted for by paths in the model.

Figure 2 presents the standardized solution to the final model with correlated variances/unique variances added, S-B *χ*^2^(35) = 131.52, *p *< .001, Robust CFI = .92, RMSEA = .07, 90% CI = [.05, .08]. The model reproduced observed correlations with an average absolute standardized error of .03. All parameters estimated in the model were significant at the .05 level or better. Supplementary Table S7 presents the correlation matrix, variances, and standardized residual for the final model.

Similar relations between maternal parenting practice factors and domains with infant behavior domains were found in the Study 2 international sample as in the Study 1 U.S. sample. The maternal dyadic factor related positively to the infant social domain; the maternal extradyadic factor and the unique variance of the mother physical domain related positively to the infant exploration and physical domains; and the infant communicated distress domain and nondistress vocalization (negative) domain related to the maternal nurture domain.

#### Infant sex

To test whether the final international sample model fit equally well for mother-daughter and mother-son dyads, a series of nested multisample models was constructed that sequentially introduced constraints on the measurement model path coefficients and covariances (61). All factor loadings, factor variances, and unique variances were constrained to be equal. A preliminary test in which no parameter estimates were constrained to be equal fit the data, *χ*^2^(70) = 166.88, *p *< .001, CFI = .92, RMSEA = .07, 90% CI = [.05, .08], suggesting that more restrictive models were appropriate. In testing the series of nested multisample models, a consistent pattern emerged in which one path coefficient (the infant nondistress vocalization domain to the mother nurture domain) differed for mothers of female infants and mothers of male infants; there were no differences in any other parameter estimates. In the most rigorous test in which all parameters except this one path coefficient were constrained to be equal, an adequate fit was achieved, *χ*^2^(100) = 191.20, *p *< .001, CFI = .93, RMSEA = .05, 90% CI = [.04, .06]. The difference in *χ*^2^ between this model and the model that imposed no invariance constraints was not significant, *χ*^2^(30) = 24.32, *p *= .76, suggesting that, apart from the one path coefficient, imposing invariance across the measurement model, path coefficients, and covariances had no deleterious effects on model fit. The one path coefficient which showed an infant sex difference was the path from the infant nondistress vocalization domain to the mother nurture domain (standardized coefficient = −.04, *p *= .41, for mothers of female infants and −.21, *p *< .001, for mothers of male infants): nondistress vocalizing in male infants was associated with less maternal nurturing, but no such relation existed in female infant-mother dyads.

### Study 2: discussion

Mothers' parenting young infants in several samples from around the world has a higher-order bifactorial (dyadic and extradyadic) structure that is expressed in six domains [nurture, physical, social, didactic, material, and language (4)]. Infant behaviors aggregate into five domains (physical, social, exploration, nondistress vocalization, and distress communication). In terms of their interactions, mothers who engage in more dyadic focus have infants who engage in more social exchanges with their mothers; and mothers who engage in more extradyadic focus and mothers who encourage their infants' physical development more have infants who explore their environments more and who exhibit higher levels of motor development. Finally, mothers whose infants communicate distress more and vocalize nondistress less nurture their infants more. The overall structure of parenting infants in several international samples largely obtains for mothers with daughters and sons. The General Discussion sets these results in broader empirical and theoretical contexts.

Cross-national research usually compares two (or just a few) national locales. However, the number of rival explanations of a common phenomenon can be reduced when the number of samples compared is increased (63), so the larger the number of national samples studied the more compelling is the conclusion that any observed generic findings (here about the nature and structure of mother-infant interactions) may be robust and internationally common. For Study 2, mothers and infants were recruited from 9 South American, North American, European, African, Middle Eastern, and East Asian countries that also varied on dimensions of possible national comparison that included, for example, economic, educational, ecological, and climatic factors (64). Despite this variation, a common structure of mother-infant interaction emerged.

## General discussion

The central aims of these studies were to explore relations of two maternal parenting practice factors and six domains to five infant behavioral domains in U.S. and international samples and assess whether models of mother-infant interactions were moderated by infant sex. Previous work with both the U.S. and 9 international samples supported a 2 factor/6 domain model of maternal parenting practices (4). Here, the maternal parenting practice factors and domains related in systematic, specific, and similar ways to infant behavior domains in U.S. and international samples with only three of many possible associations moderated by infant sex. Developmental science benefits from examining the perspectives of parent, child, and context simultaneously. The parental perspective provides the vital social circumstances for child care; the child perspective provides an indispensable basis for assessing the impact of caregiving; and the contextual perspective provides the ideals and practices of society or nation that embeds parent and child. Here we discuss relations between the maternal factors and domains and infant domains, strengths and limitations of the U.S. and international studies, the role of infant sex in mother–infant interactions, as well as theoretical conclusions and clinical implications of this work.

### Mother-Infant interaction: associations and specificities

The design of the current studies was cross-sectional, and so strictly speaking their results are fittingly interpreted in terms of associations between mothers and infants and do not untangle direction of “effects” or imply causation between mothers and infants. However, the parent-infant relationship is in many respects regarded as asymmetrical, acknowledging the predominant role mothers play in guiding interactions with their young infants (65), and we hypothesized some relations from mothers to infants. We therefore first discuss mother-infant “effects” in the data but also point to some clear reciprocal infant “effects” on mothers. Furthermore, in this discussion we identify and elaborate specificities revealed in these mother-infant interaction data. Of course, inability to specify direction of effects in no way subtracts from important conclusions about structure, association, correspondence, and specificity in mother-infant relationships. The specific concurrent correspondences begin to characterize important mutual influences that mother and infant exert on one another from an early period in the infant's life.

#### Mother-infant effects

Although parental genes contribute to infant proclivities and abilities in different domains, all prominent theories of development, such as relational systems, judge experience in the world as either the principal source of individual growth or as a major contributing component (2). Parents (and other caregivers) furnish and shape infants' experiences and directly influence infant development by the attitudes they hold and by the actions they exhibit. Evidence for heritability neither negates nor diminishes equally compelling empirical evidence for the direct short- and long-term influences of parent-provided experiences in infant development (3, 66).

These companion studies revealed two prominent associtions between mothers' parenting practices and their infants' behaviors. The first association was mothers' dyadic factor in relation to infant social behaviors with mother. One central task of infancy is achieving wholesome emotional and social development. Mothers help their infants to reach this goal by forging close interpersonal relationships. Thus, mothers engaging in dyadic forms of interaction is integral to parenting and infant socioemotional successes (67–69). Parental warmth and emotional support (a dyadic focus) appear to be important for infant's social exchanges and for children's future socioemotional competencies. The second set of associations that emerged were mothers' extradyadic factor and their encouragement of infant physical development in relation to infant exploration of the immediate physical environment and infant physical development. A second central task of infancy is accommodating to and coming to understand the material world outside the dyad. Mothers' extradyadic parenting practices and their promotion of infant physical development scaffold infants' exploratory behaviors and physical development. Being introduced to and beginning to negotiate the world outside the dyad are similarly significant developmental briefs for young infants.

#### Infant-mother effects

These analyses also revealed two prominent expected associations of infants' behaviors with their mothers' parenting practices. First, infants' distress communication was associated with maternal nurturing; that is, infants who fretted and cried had mothers who appropriately attended to them. It is less likely that mothers who nurture have infants who communicate more distress and more likely that infants who communicate distress have mothers who nurture. This infant-effects interpretation is supported by a three-culture study that examined and compared coded maternal responsiveness to infant activities during home-based naturalistic interactions in New York City, Paris, and Tokyo; it revealed that mothers do indeed respond to their infants' vocalizing distress with nurturance [(70); see also (20, 21)]. Nonetheless, confirmation of direction of effects will depend on sequential analyses, longitudinal designs, and experimental investigations. Second, infants' nondistress vocalization was negatively related to maternal nurturing; that is, mothers whose infants were vocalizing nondistress (cooing, babbling, and the like) refrained from engaging in similar nurturing activities with their infants. These infant effect findings accord with many examples that populate the developmental science literature. Notoriously, infant physiognomy attracts adults (71), and Lorenz (72) contended that facial features of “babyishness” universally provoke adults to reflexively express solicitousness towards infants.

The dyadic-extradyadic balance that characterizes the mother-infant relationship likely has meaningful and far-reaching consequences in the life of the child as the dynamic systems perspective posits that reciprocity between mother and infant specifically facilitates higher-level forms of interaction. To the extent that mothers effectively support and promote both affiliative and exploratory goals for their infants, infants' chances to develop both socioemotional and mental adaptive competencies are improved. These two equally vital systems are present at a surprisingly early point in the development of the mother-infant relationship and across an equally surprising diversity of national contexts.

### National and international studies of parenting and infancy: commonalities and specificities

These studies fall into the category of international developmental science, and the “story” of that approach to understanding human ontogeny is at base one of similarities and differences, universals and specificities (6, 12). The story that emerged from these studies is no different. Admittedly, “universal” is likely never truly universal (there being some exception somewhere, and proving a universal is impossible), and “specific” is likely never truly specific (there being some commonality somewhere, and proving a specific is likewise impossible). Nonetheless, noteworthy nomothetic and some idiographic lessons emerged in these studies.

#### On commonalities

Two sorts of broad commonalities emerged from these studies, one having to do with conceptually corresponding mother-infant associations and a second having to do with infant sex. Across samples in these studies, maternal parenting practices related to corresponding infant behaviors: Mothers who encouraged infants in more social activities had infants who engaged in more social activities, mothers who didactically encouraged their infants had infants who explored their environments more, mothers who fostered their infants' physical development more had infants who were physically more developed, and infants who vocalized distress more had mothers who nurtured their infants more. Such universals in parenting and infancy are supported by several arguments. First, there may be special and exacting constraints and demands in parenting infants that opportunistically apply universally. That is, certain parenting practices could recur across (even very different) contexts on account of common determinants of parenting in, say, factors endemic to evolution, to biology, to social history, or to children. Universal characteristics of parenting may be instinctual to a parenting “stage” in the human life cycle. It could be in the nature of being a human being—as much as a parent—to optimize the success of one's offspring and thereby to ensure the survival of one's genes (8, 73). Maternal hormones and the maternal nervous system may have evolved to treat and respond to human infants in some uniform ways (14, 20, 21, 74, 75). A universalist position also points to the shared environment as cause of uniformity in parenting; that is, certain economic or ecological factors are common even to different national samples on account of worldwide historically converging and homogenizing patterns of modernization, urbanization, Westernization, migration, or dissemination *via* media, and they cumulatively contribute to the deconstruction of many traditionally differentiated cultural patterns. On purely empirical grounds, moreover, the groups participating in these studies also created the possibility of identifying generalities of childrearing. The locales studied are more alike than not in terms of modernity, urbanity, economics, politics, living standards, even ecology and climate, and therefore it was possible to recruit roughly equivalent sociodemographic samples. Families were typically nuclear in organization; mothers normally the primary caregiver in the family setting; and parents shared many of the same larger and long-term goals for their children, notably physical health, social adjustment, mental achievement, and economic security. Last, by virtue of their helplessness or those “babyish” characteristics mentioned earlier, which are of course structurally universal, infants may elicit common patterns of interaction from their caregivers (71, 72, 76–78). Separately or collectively, these several evolutionary, biological, historical, and interpersonal forces likely engender some similarities in parenting and in infant development (79). Which specific force or forces these converging patterns reflect is difficult, if not impossible, to determine.

The second commonality to emerge from these studies had to do with infant sex. It is important to attend to sex similarities and differences in infancy for a variety of reasons, not the least of which are that even small differences in sexed patterns of development or treatment of infants likely cumulate over time; moreover, even if female and male infants are treated similarly the two could still experience similar environments or interpret similar experiences differently (13). Much of what is known about parenting infants and infant development with respect to sex comes from small samples and single locales in the minority world of WEIRD countries where a long-standing tradition has touted sex differences. However, the keen and consistent historical attention paid to sex in cultural, parenting, and developmental sciences has tended to substantiate surprisingly few practicable sex differences (13). Indeed, on the basis of more than 45 meta-analyses Hyde (80, 81) advocated a more general “gender similarities” hypothesis. In the current two studies, equivalent numbers of female and male infants were recruited into each sample, and in support of that similarities hypothesis analyses of associations of the six factors and two domains of maternal parenting practices with the five infant behavioral domains revealed only three moderation effects by infant sex of 80 possible mother-infant relationships (<4%). In accord with the gender similarities hypothesis, female and male infants appear to differ relatively little in their interaction experiences.

#### On specificities

A prevailing assumption in parenting studies is that the overall level of parenting affects children's overall level of functioning. This position has been challenged by a more differentiated view that asserts specificity in parent-offspring interactions (82). That is, developmental relationships between parents and infants are not generalized, but are specific. As mentioned, only conceptually related mother–infant correspondences proved to be common across diverse international samples, despite considerable variation in many national traditions and situations, attitudes and actions. Domain-specific mutual correspondences in mother–infant interaction patterns appear to be widespread and similar across international samples. By contrast, there was little correspondence between maternal parenting practices and infant behaviors that were not conceptually related. In other (concrete and contrasting) words, mothers who engage in extradyadic practices more with their infants do not have infants who necessarily engage them more socially, and mothers who engage their infants more dyadically do not necessarily have infants who explore their environments more. The infants in these studies are only 5½ months of age, barely beyond *fetus ex utero*; so, specific mother–infant attunement is also fast developing.

It has been thought by some that differences among children's common home environments within the normal species range have no effect on children's outcomes [(83); see also (84)]. But parenting and infant behaviors alike vary, and patterns of associations between them suggest that variations within the normal range in particular kinds of parenting practices are associated with variations within the normal range in particular kinds of infant behaviors. Infants and mothers tend to show attunement and specificity with one another, and increasing evidence suggests that specific (rather than general) parental activities relate concurrently (and predictively) to specific (rather than general) aspects of infant performance or competence (82). These findings support the *specificity principle* which holds that specific experiences at specific times exert specific effects over specific aspects of child development in specific ways (3, 6, 20, 82). Developmental scientists and theoreticians today do not ask whether caregiving affects child development, but which parent-provided experiences affect which aspects of child development when and how, and they are interested also to learn the ways in which individual children are so affected, as well as the ways individual children affect their own development. To detect regular relations between antecedents in parenting, experience, and environment on the one hand and outcomes in infant and child characteristics on the other, we need to seek and to find precise and specific combinations of independent and dependent variables. In this light, parenting and infancy alike are each best conceptualized as multivariate, modular, and specific in nature.

### Limitations of these studies

The design and analyses of these studies and the generalizability of the results are constrained by main terms of the Specificity Principle—setting, person, time, process, and outcome (3, 6, 20, 82). These two studies focused on specific activities (the maternal parenting practice and infant behavior indicators, domains, and factors) occurring in specific types of interactions (open, naturalistic) in specific settings (the familiar home) under specific conditions (infants in awake, sated, and alert states with dyads alone) in specific people (primiparous mothers and their 5½-month infants) in (nine) specific countries at a particular point in historical time. In consequence, the findings apply and might generalize specifically to similar activities, interactions, settings, persons, conditions, people, countries, and times. Investigations of other parenting practices and other infant behaviors in other situations under other conditions at other times by other categories of mothers with children of other ages could result in similar or different patterns of results. For example, mother-infant interactions could change dramatically in the context of multiple caregivers and multiple infants all present at the same time. Additionally, fathers actively engage in caregiving and are acknowledged to make independent contributions to children's development (85, 86). Furthermore, samples from 9 different countries were recruited, including two in South America, one in North America, three in Europe, one in the Middle East, one in Africa, and one in East Asia; however, no claims are made that they are representative of their nations or of the world's cultures. All these considerations naturally constrain the generalizability of the parenting practice and infant behavior domain structures as well as the patterns of practice-behavior relations reported here.

Due to the complexity of the models and the small sample sizes in some countries, we were not able to test meaisrement invariance of the model across countries. Our strategy to combine all countries in a single model answers whether the model is generalizable in a more heterogeneous multi-country sample, but it does not tell us whether the model holds in each and every country.

Etic constructs consist of accounts, descriptions, and analyses of activities that apply broadly across cultures and are expressed in terms of conceptual schemes and categories that are regarded as meaningful and appropriate by the broad community of scientific observers. By contrast, emic constructs consist of accounts, descriptions, and analyses expressed in terms of conceptual schemes and categories that are regarded as meaningful and appropriate by members of a particular community. The maternal parenting practices and infant behaviors studied here are likely etic, but their emic connotations could still differ across samples (64, 79). The everyday maternal parenting practices and infant behaviors operationalized and observed in these studies were representative of prominent and common interactions of new mothers with their young infants and were identical across the different cultural settings, so their comparability was assured. Nonetheless, understanding the relation between activity and meaning depends on context as well as the unit of analysis and the level of abstraction chosen for analysis.

### Implications for theory and practice

These studies aimed to learn more about human parenting during the significant period of the mother-infant dyad's initial mutual accommodation in the middle of the first year of the infant's life by recording, coding, analyzing, and comparing specific observable parenting practices of mothers and behaviors of infants across diverse samples. Empirical and clinical implications of this work would be twofold. First, empirically, studies of parenting, infancy, and mother-infant interactions have been undertaken at microanalytic and macroanalytic levels. Here, individual maternal parenting practices and infant behaviors were coded microanalytically, and domains and factors of mother-infant interactions were examined. By assessing these foundational in-the-moment building blocks of mother-infant interaction and how they are structured and manifest across international samples, these studies provide insights for future students of parenting, infancy, and family theory as well as international developmental science. Among many open questions would be to evaluate predictive associations of these parenting factors and domains for later child development.

Second, this close analysis of everyday behaviors also has relevance for the intersection of cognitive and clinical science, as such naturalistic tasks as basic caregiving require the coordination of multiple cognitive faculties whose deconstruction could reveal which specific processes underlie which specific caregiving shortcomings (87, 88). How mother-infant interactions vary in atypical populations may have important implications for therapeutic diagnosis and treatment.

## Conclusions

These studies suggest that maternal parenting practices and infant behaviors are structured and that mother-infant interactions are characterized by conceptual correspondences consistent with notions of commonality, specificity, and modularity (6, 89). Parenting and infancy are common and modular in the senses that the two factors and six domains in mothers relate to separate developmental domains in infants, and they are specific in the sense that maternal dyadic activities relate to infant social activities and maternal extradyadic activities relate to infant exploration and physical development.

Certain enduring psychological characteristics might arise early in life, and the nature of parent-infant interaction is thought to contribute to individual development and cultural variation. As a result, studies of parenting, infancy, and mother-infant relationships have often been undertaken in attempts to address questions about caregiving, the origins and early development of individuals, and the influences of culture. Moreover, assumptions about the specificity and commonality of parenting, infants, and relationships between parents and infants may be advantageously tested within the context of international developmental research.

Mothers across different national samples showed some striking similarities in interacting with their infants. These converging patterns might reflect inherent attributes of caregiving (at least in industrialized and developed societies) or the historical convergence of parenting styles or the increasing prevalence of homogeneous childrearing patterns. They may also be instigated by infants themselves. In the end, different people (presumably) wish to promote similar general competencies in their offspring, and they do so in some manifestly similar and specialized ways.

## Data Availability

All data and materials for these studies may be accessed, upon reasonable request and for legitimate scientific purposes, by contacting the corresponding author.
