# Supplementary material for: The nature and structure of maternal parenting practices and infant behaviors in U.S. national and international samples
Source: Front Child Adolesc Psychiatry. 2023 May 26;2:1124037. doi: 10.3389/frcha.2023.1124037 (PMC10281019; doi:10.3389/frcha.2023.1124037)
Supplement: Supplementary file 1 [file Datasheet1.docx]

**The Nature and Structure of Maternal Parenting Practices and Infant Behaviors in**

**U.S. National and International Samples**

**Supplementary Material**

**Justification for the Focus on Mothers**

Mothers were chosen as the parent figure for these studies because almost all mammalian species are matrilocal (1), and most animal and human families matriarchal. Theoreticians distinguish between bringing a new individual into the world (childbearing) on the one hand and rearing an existing individual (childcaring) on the other. Whereas species lower in the phylogenetic hierarchy are principally bearers of young, mammals are devoted child carers (2), and early maternal care is more common than paternal care among land mammals (3), where males provide care in fewer than 5% of species (4). Cross-cultural surveys and meta-analyses alike attest to the central role that mothers play in infant development (5-7). Furthermore, mothers and fathers do not share the same parenting investment strategies (2, 8), and the maternal role is better articulated and defined than is the paternal role (9). According to Konner (10), there is not a single traditional society in which fathers devote more time to childcare than mothers, and mothers participate in childrearing activities at significantly higher rates than do fathers (11) where, on average, mothers spend between 65% and 80% more time than do fathers in direct one-to-one interaction with their infants (8). Given societal dictates that mothers are also ultimately responsible for their young children’s health and well-being, females have traditionally become more expert caregivers than males (12, 8). Many societies have witnessed increases in the amounts of time fathers spend with their children; in reality, however, fathers typically assume little direct responsibility for infantcare and rearing and are primarily helpers (8). For these reasons, theorists, researchers, and clinicians have historically concerned themselves preponderantly with mothering, rather than fathering. There is a greater body of information about mothers and infants, than about fathers, siblings or other relatives, or nonfamilial caregivers, and we wished to make contact with that literature.

**Preliminary Analyses and Analytic Plan**

Prior to all analyses, univariate distributions for all domain scores were examined for normality and outliers (13, 14), and the distance of each case to the centroid was evaluated to screen for multivariate outliers (15). To approximate normality and reduce the number and influence of outliers, variables were transformed as needed; the same transformations were applied to the U.S. and international data; and all analyses were conducted on transformed data.

The best-fitting structures of maternal parenting practices and infant behaviors were evaluated in multiple steps using structural equation models (SEM). All SEMs were fit using maximum likelihood functions and followed the mathematical models of Bentler and Weeks (16) as implemented in EQS (17). Model fit was assessed using multiple, convergent indices, including the robust Satorra-Bentler (18, 19) scaled χ^2^ statistic, robust comparative fit index (Robust CFI) (20), and the root mean square error of approximation (RMSEA) (21) and its 90% confidence interval. To enhance the cross-validation adequacy of models, the Akaike Information Criterion (AIC) (22, 23) was monitored for its decreasing value in all nested models. Cutoff values close to .95 and .06 for CFI and RMSEA, respectively, are indicative of a relatively good fit between a hypothesized model and the observed data (24).

Prior to fitting any SEM, bivariate plots were inspected to confirm that domains were linearly related and that no curvilinear effects existed between pairs of domains. In the course of fitting SEMs, Mardia (25) coefficients of multivariate kurtosis and cases that contributed most to those estimates as well as the stability of parameter estimates and cases that contributed disproportionately to those estimates were evaluated. In the SEMs, univariate measures of kurtosis and normalized estimates of Mardia’s multivariate coefficients indicated no significant problems of nonnormality.

Re-specifications of models were planned using the following strategy: First, to simplify the model, all nonsignificant paths were dropped based on the multivariate Wald statistics. When all remaining estimated parameters in the model were significant, single paths were then added sequentially to the model. At each step, the standardized residual covariance matrix was studied, together with Wald and Lagrange multiplier tests, and potential paths were evaluated for their theoretical plausibility and empirical probability (26, 27). Models that are constructed by sequentially testing the addition and removal of paths, even when selection of paths is guided by theoretical relevance and plausibility, tend to exploit ungeneralizable relations in the data. For this reason, final modified models are treated as having tentative explanatory value, suggesting relations for testing in future research.

***Study 1: U.S. Sample Mother-Infant Interaction Model.*** The hypothesized mother-infant interaction model contained -- other than the paths in the mothering measurement model (see 28) -- direct paths from mother dyadic focus to infant physical, social, and nondistress vocalization, from mother extradyadic focus to infant physical, exploration, and nondistress vocalization, and from infant distress communication to dyadic focus. The *a priori* model did not fit the data, S-B χ^2^(40) = 140.27, *p* < .001, Robust CFI = .83, RMSEA = .08, 90% CI = (.07, .10). Wald tests suggested dropping several paths, and Lagrange multiplier tests suggested adding paths.

***Study 2: International*** ***Sample*** ***Mother-Infant Interaction Model.*** The mother side of the international samples model was derived in a previous analysis (see 28). The infant side of the model was carried over from Figure 1 without the 2 pairs of correlated infant variances/unique variances. When this mother-infant structural model was fit to the international data set, all factor loadings and path coefficients were found to be significant at the .05 level or better. However, the overall χ^2^ test and the RMSEA indicated that the model left significant covariation unexplained, S-B χ^2^(38) = 230.66, *p* < .001, Robust CFI = .83, RMSEA = .09, 90% CI = [.08, .10]. Lagrange multiplier tests identified three pairs of correlated infant variances/unique variances: Distress communication (negative)with social, χ^2^(1) = 34.65, *p* < .001, with exploration, χ^2^(1) = 28.93, *p* < .001, and with nondistress vocalization, χ^2^(1) = 40.92, *p* < .001. These three covariances were allowed because it is incompatible that distressed young infants simultaneously engage socially with their mothers, explore the environment, and vocalize nondistress.

**Supplementary Table S1** Maternal parenting practice domains, definitions of constituent indicator parenting practices, interim variables, final indicator variables, and mean standard scores of final variables.

| Domains | Definitions of Constituent Indicators | Interim Variables | Final Indicator Variables | Mean Standard Scores of Final Variables |
| --- | --- | --- | --- | --- |
| Nurture | *Feed*: Attempting to give the infant liquid or solid foods by cup, bottle, breast, or spoon | Duration of feed | Sum of durations of feed and burp/wipe | Mean of standard scores of total duration of feed/burp/wipe, duration of bathe/diaper/dress, groom/other, and duration of hold |
|  | *Burp/Wipe face or hands*: Attempting to burp the infant in connection with a feeding or wiping the infant’s face, hands, or clothing at any time | Duration of burp/wipe |  |  |
|  | *Bathe*: Washing and drying the infant’s body and/or hair | Duration of bathe | Sum of durations of bathe, check/change diaper, dress, groom, and meet other health needs |  |
|  | *Check/Change diaper*: Checking if the infant needs a diaper change or changing the diaper | Duration of check/change diaper |  |  |
|  | *Dress*: Removing or putting an article of clothing on the infant | Duration of dress |  |  |
|  | *Groom*: Engaging in a practice designed to enhance the infant’s appearance (e.g., combs hair) | Duration of groom |  |  |
|  | *Meet other health needs*: Attending to other health needs of the infant (e.g., wipes or suctions the infant’s nose; gives medicine from a dropper or medicine spoon) | Duration of meet other health needs |  |  |
|  | *Hold*: Supporting some or all of the infant’s weight with the mother’s body | Duration of hold | Duration of hold |  |
| Physical | *Physically encourage to sit:* Placing the infant in a sitting position in which the infant’s back is not leaning against a firm surface | Proportion of consecutive 10-min time units in which physical encouragement to sit was observed | Mean proportion of consecutive 10-min time units in which physical encouragement to balance (sit or stand) was observed | Mean of mean proportions of encourage balance and encourage movement |
|  | *Physically encourage to stand:* Placing or holding the infant in a standing position so that there is some weight supported by the infant’s straightened legs | Proportion of consecutive 10-min time units in which physical encouragement to stand was observed |  |  |
|  | *Physically encourage to roll:* Physically assisting the infant to roll over | Proportion of consecutive 10-min time units in which physical encouragement to roll was observed | Mean proportion of consecutive 10-min time units in which physical encouragement  to move (roll, crawl, or step) was observed |  |
|  | *Physically encourage to crawl*: Physically assisting the infant to move forward (on the belly or on hands and knees) by moving the infant’s arms and/or legs or by pushing rump or feet from behind | Proportion of consecutive 10-min time units in which physical encouragement to crawl was observed |  |  |
|  | *Physically encourage to step:* Holding the infant in a standing position and then moving the infant’s body to simulate stepping movements | Proportion of consecutive 10-min time units in which physical encouragement to step was observed |  |  |
| Social | *Encourage attention to mother*: Attempting to draw the infant into face-to-face social interaction with herself physically by intentionally moving her face toward the infant or moving the infant toward her face or verbally by making very specific comments about herself that are clearly designed to capture the infant’s interest (pauses of 2 ss or longer are coded as terminations of an ongoing practice) | Frequency of encouraging attention to mother  Duration of encouraging attention to mother | Mean standard score of frequency and duration of encourage attention to mother | Mean of standard scores of encourage attention to mother, social play, and express affection |
|  | *Social play*: Directing verbal or physical behaviors to the infant, the purpose of which appears to amuse the infant (i.e., to elicit smiles, positive nondistress vocalizations, laughter, or motoric excitement in the context of a primarily social dyadic interaction). Coding is discontinued when the mother has not interacted for 3 s and when she is no longer oriented to the infant and poised to continue the exchange; pauses of any duration when the mother clearly remains poised to continue are coded as part of the social play sequence. The types of exchanges coded as social play are: (a) physical contact with a fun-like quality (e.g., tickling); (b) introducing the element of surprise, suspense, or quick release of stimuli (e.g., peek-a-boo); (c) singing to the infant; and (d) playing a game that involves physical manipulation of the infant’s body (e.g., pattycake) | Frequency of social play  Duration of social play | Mean standard score of frequency and duration of social play |  |
|  | *Express affection.* Expressing affection or positive evaluation to the infant either physically (e.g., kissing, patting, stroking, or caressing) or verbally (using explicit phrases denoting praise or endearment) | Frequency of expressing affection Duration of expressing affection | Mean standard score of frequency and duration of expressing affection |  |
| Didactic | *Encourage attention to object*: Physically moving the infant or an object so that the infant can see or touch it or verbally referring to an object or an object-related event or activity that is no more than 12 feet from the infant (pauses of 2 s or longer are coded as terminations of an ongoing practice) | Frequency of encouraging attention to object  Duration of encouraging attention to object | Mean standard score of frequency and duration of encouraging attention to object | Mean standard score for encourage attention to object |
| Material | *Quantity of objects provided infant*: The number of toys, books, and household objects that are within the infant’s reach | Variety of objects provided: The number of different objects that are within infant reach during the total observation  Density of objects provided: The mean number of objects within infant reach per consecutive 5-min time unit  Consistency of objects provided: The number of consecutive 5-min time units in which any object was within infant reach | Mean standard score of the quantity (variety, density, and consistency) of objects provided | Mean of standard score for quantity of objects |
|  | *Quality (responsiveness) of objects provided infant*: Ratings of all toys, books, and household objects within reach of infant on four dimensions: moving parts, change in shape or contour, noise production, and reflected image | Responsiveness of objects: Mean of sums of ratings for moving parts, change in shape or contour, noise production, and reflected image for each toy, book, and household object within infant reach  Number of highly responsive objects: Number of toys, books, and household objects within infant reach that has a sum of ratings for moving parts, change in shape or contour, noise production, and reflected image *>*12 (on a scale of 4–16)  Proportion of highly responsive objects: Proportion of toys, books, and household objects within infant reach that has a sum of ratings for moving parts, change in shape or contour, noise production, and reflected image *>*12 (on a scale of 4–16) | Mean standard score of quality (responsiveness, number of highly responsive, and proportion of highly responsive) of objects provided | Mean of standard score for quality of objects |
| Language | *Speech to the infant:* Mother’s speech steam directed to the infant in either adult- or infant-directed tones, including vocalizations of syllables, parts of words, words, and singing, was coded continuously. Changes and pauses lasting less than 1 s were not recorded, and vocalizations between pauses lasting more than 3 s were recorded as different | Frequency of vocalizations  Duration of vocalizations | Mean standard scores of frequency and duration of speech to the infant | Mean standard score for speech to the infant |
|  |  |  |  |  |

*Note*. Reproduced from (28).

**Supplementary Table S2** Infant behavior domains, definitions of constituent indicators of infant behaviors, interim variables, final indicator variables, and mean standard scores of final variables.

| Domains | Definitions of Constituent Indicators | Interim Variables | Final Indicator Variables | Mean Standard Scores of Final Variables |
| --- | --- | --- | --- | --- |
| Physical | *Sit*: Infant is placed in initial sitting position, ability to maintain and control sitting balance is evaluated, with control lasting a minimum of 30 continuous s. The rating scale contains 8 levels plus “Not coded” (no opportunity to observe) from Level 1 = Sits with back rounded and head unsteady (bobs, leans to side, falls forward) when fully supported in inclined sitting position—adult’s lap, infant seat, and so forth to Level 8 = Rotates from prone position to a balanced sitting position with weight on buttocks and without assistance | The score for sit was converted to its equivalent developmental level expressed in months | Balance is the highest developmental level score for sit observed in 5 consecutive 10-min time units | Mean of balance and movement |
|  | *Prelocomotion, Upper Body*: The prone infant lifts the head and shoulders and/or extends the arms, lasting a minimum of 30 continuous s unless otherwise noted. The rating scale contained 5 levels plus “Not coded” (No opportunity to observe) from Level 1 = The prone infant lifts head and shoulders for 5+ seconds, arms not used as primary support to Level 5 = The prone infant, up on extended arms and able to reach with one arm, shifting weight and remaining balanced with well-coordinated movements | Upper body prelocomotion, lower body prelocomotion, and locomotion scores were initially converted to their equivalent developmental levels expressed in months. Then a movement score expressed in months was computed for each of the 5 10-min observation periods as the highest of the developmental level scores for upper body prelocomotion, lower body prelocomotion, and locomotion | Movement is the highest movement score expressed in months observed in 5 consecutive 10-min time units |  |
|  | *Prelocomotion, Lower Body*: The prone infant extends/lifts hips, bends knees, and supports weight on knees and lower legs, lasting a minimum of 30 continuous s. The rating scale contained 4 levels plus “Not coded” (No opportunity to observe) from Level 1 = The prone infant extends legs with hips resting on supporting surface to Level 4 = The prone infant supports weight on knees and lower legs and off thighs (full crawl position, with or without movement) |  |  |  |
|  | *Locomotion:* The infant displays “deliberate” or nonaccidental, unassisted movement in any direction lasting a minimum of 30 continuous s, with or without a noted “goal.” The rating scale contained 11 levels plus “Not coded” (No opportunity to observe) from Level 1 = The infant lifts legs when in a supine position, with or without attempts to grasp the feet or legs, the arms and legs are active to Level 11 = The infant actively creeps across the room |  |  |  |
| Social | *Look at mother*: The infant looks at the mother’s face or head. Focused fixation must be evident. An active behavior component often accompanies clear and focused fixation (e.g., brightening of the face, widening of the eyes, stilling, increased motor excitement, positive vocalizations, or reaching). A change in fixation is coded after the infant has looked away from target for 1 s | Frequency of looking at mother  Duration of looking at mother | Mean standard score of frequency and duration of looking at mother | Mean of standard scores for looking at mother, smiling, and alert expression |
|  | *Smile*: The infant emits a clear, unambiguous smile. The corners of the infant’s mouth are extended outward and upward; the eyes “brighten” and are focused; and the eyebrows are relaxed or raised | Frequency of smiling  Duration of smiling | Mean standard score of frequency and duration of smiling |  |
|  | *Alert expression*: The infant’s face poses as expressions of interest, concentration or seriousness, questioning looks, and wide-eyed alertness | Duration of alert expression | Mean standard score of duration of alert expression |  |
| Exploration | *Look at object*: The infant looks at any discrete object or body part other than a face that is within a radius of 12 feet. Focused fixation must be evident. An active behavior component often accompanies clear and focused fixation (e.g., brightening of the face, widening of the eyes, stilling, increased motor excitement, positive vocalizations, or reaching). A change in fixation is coded after the infant has looked away from target for 1 s | Frequency of looking at object  Duration of looking at object | Mean standard score of frequency and duration of looking at object | Mean of standard scores for looking at object, touching object, mouthing object, extent of exploration, and efficiency of exploration. |
|  | *Touch object*: The infant actively and purposefully handles an object by grasping and moving it (e.g., lifting, waving, banging, dropping, rotating, and turning) or by directly exploring the object using the palm or fingers of the hand (e.g., patting, rubbing, squeezing, and fingering). A change in behavior is coded when the new behavior has lasted for 1 s | Frequency of touching object  Duration of touching object | Mean standard score of frequency and duration of touching object |  |
|  | *Mouth object*: A discrete object other than a pacifier or bottle is in contact with the infant’s mouth. A change in behavior is coded when the new behavior has lasted for 1 s | Frequency of mouthing object  Duration of mouthing object | Mean standard score of frequency and duration of mouthing object |  |
|  | *Extent of exploration*: The amount of touching or mouthing an infant does of toys, books, or household objects within reach | Variety of objects explored: The number of different objects explored during the total observation.  Density of objects explored: The mean number of objects explored per 5-min time unit.  Consistency of objects explored: The number of consecutive 5-min time units in which any object was explored | Mean standard score of variety, density, and consistency of objects explored |  |
|  | *Efficiency of exploration*: The proportion of toys, books, or household objects within reach that an infant touches or mouths | Proportion variety: The proportion of available objects explored during the total observation.  Proportion density: The mean proportion of available objects explored per 5-min time unit.  Proportion consistency: The proportion of consecutive 5-min time units containing available objects in which exploration occurred | Mean standard score of proportion variety, proportion density, and proportion consistency |  |
| Nondistress vocalization | *Non-distress vocalization*: Any positively or neutrally toned vocalization that is clearly audible, including babbling, cooing, laughing, vocal play, shrieking, and sighs or grunts not indicative of distress. Nondistress vocalizations of any duration are coded; brief pauses in nondistress vocalization of less than 1 s are not recorded | Frequency of nondistress vocalization  Duration of nondistress vocalization | Mean standard score of frequency and duration of nondistress vocalization | Mean standard score for non-distress vocalization |
| Distress  communication | *Negative facial expression*: Displays of distressed, angry, disgusted, or frowning expression, characterized by at least two of the following: mouth opened and lips stretched horizontally, mouth closed and lips pressed together, an inward and downward pressing of the bridge of the nose, and one or more horizontal furrows across the forehead, especially just above the eyebrows | Frequency of negative facial expression  Duration of negative facial expression | Mean standard score of frequency and duration of negative facial expression | Mean of standard scores of negative facial expression and distress vocalization. |
|  | *Distress vocalization*: Vocalizations that indicate protest, complaint, anger, or upset, as indicated by vocal quality, facial expression, or other negative behaviors (e.g., intense squirming, back arching). Vocalizations of any duration are coded; brief pauses in vocalization of less than 1 s are not recorded | Frequency of distress vocalization  Duration of distress vocalization | Mean standard score of frequency and duration of distress vocalization |  |

**Supplementary Table S3** Intercoder Reliabilities of Maternal Parenting Practice and Infant Behavior Domains by Country

|  | Reliability Index | Argentina  (*n*s = 16-26) | Belgium  (*n*s = 18-34) | Brazil  (*n*s = 5-14) | France  (*n*s =10-13) | Israel  (*n*s = 5-10) | Italy  (*n*s = 11-20) | Japan  (*n*s = 9-17) | Kenya  (*n*s = 5-11) | United States  (*n*s = 69-99) |
| --- | --- | --- | --- | --- | --- | --- | --- | --- | --- | --- |
| 1. Maternal Parenting Practices | | | | | | | | | | |
| Nurture | Kappa | .89 | .90 | .88 | .91 | .79 | .91 | .91 | .92 | .91 |
| Physical | ICC | .98 | .59 | .66 | .62 | .71 | .88 | .56 | .90 | .61 |
| Social | Kappa | .70 | .67 | .70 | .64 | .71 | .64 | .71 | .59 | .70 |
| Didactic | Kappa | .71 | .76 | .74 | .80 | .74 | .67 | .78 | .65 | .73 |
| Material | ICC | .90 | .82 | .89 | .80 | .86 | .89 | .76 | .81 | .89 |
| Language | Kappa | .70 | .66 | .65 | .72 | .89 | .71 | .68 | .64 | .69 |
| 1. Infant Behaviors | | | | | | | | | | |
| Physical | ICC | .96 | .97 | .94 | .95 | .77 | .95 | .93 | .90 | .96 |
| Social | Kappa | .62 | .56 | .60 | .62 | .63 | .60 | .54 | .55 | .62 |
| Exploration | Kappa^a^  ICC^b^ | .71  .90 | .68  .95 | .67  .95 | .68  .86 | .70  .79 | .73  .94 | .69  .93 | .70  .64 | .72  .93 |
| Nondistress  Vocalization | Kappa | .68 | .66 | .71 | .63 | .65 | .72 | .59 | .74 | .70 |
| Distress  Communication | Kappa | .67 | .65 | .65 | .76 | .71 | .70 | .72 | .64 | .68 |

^a^ Average Kappa for look at, touch, and mouth objects. ICC for the number of objects explored. Reproduced in part from (28).

**Supplementary Table S4A** Maternal Parenting Practice Domains Scores: U.S. Sample

| Maternal Parenting Practice Domains | *M* | *SD* |
| --- | --- | --- |
| Nurture | .00 | .65 |
| Physical | .18 | .13 |
| Social | .00 | .67 |
| Didactic | .00 | .91 |
| Material | .00 | .59 |
| Language | .00 | .87 |

**Supplementary Table S4B** Infant Behavior Domains Scores: U.S. Sample

| Infant Behavior Domains | *M* | *SD* |
| --- | --- | --- |
| Physical^a^ | 5.19 | .93 |
| Social | .00 | .65 |
| Exploration | .00 | .61 |
| Nondistress vocalization | .00 | .92 |
| Distress Communication | .00 | .90 |

^a^ In months.

**Supplementary Table S5** Correlation Matrix, Variances, and Standardized Residuals for Final Model (Figure 1) for the U.S. Sample

|  | 1 | 2 | 3 | 4 | 5 | 6 | 7 | 8 | 9 | 10 | 11 |
| --- | --- | --- | --- | --- | --- | --- | --- | --- | --- | --- | --- |
| 1. Mother | | | | | | | | | | | |
| 1 Nurture | **.42** | .05 | -.01 | .00 | .01 | .09 | -.02 | -.09 | .00 | .00 | -.02 |
| 2 Physical | .14 | **.03** | -.02 | -.01 | .01 | -.07 | -.03 | .02 | -.05 | -.00 | .16 |
| 3 Social | .14 | .24 | **.02** | -.02 | .05 | .02 | .00 | .03 | -.08 | .02 | .02 |
| 4 Didactic | -.18 | .23 | .39 | **.04** | .00 | -.03 | -.11 | -.02 | -.02 | -.01 | .00 |
| 5 Material | -.32 | .01 | .05 | .36 | **.35** | .01 | .08 | -.14 | -.02 | -.00 | .02 |
| 6 Language | .21 | .14 | .40 | .31 | .01 | **.76** | -.04 | -.03 | -.04 | .01 | -.08 |
| 1. Infant | | | | | | | | | | | |
| 7 Physical | -.22 | .16 | .00 | .11 | .31 | -.04 | **.00** | -.14 | -.00 | .01 | .06 |
| 8 Social | -.04 | .14 | .25 | .18 | -.14 | .16 | -.14 | **.05** | .03 | .00 | -.11 |
| 9 Exploration | -.44 | .05 | -.08 | .42 | .45 | -.04 | .30 | .03 | **.38** | .03 | .03 |
| 10 Nondistress vocalization | -.14 | -.00 | .02 | -.01 | -.00 | .01 | .01 | .17 | .03 | **.10** | .06 |
| 11 Distress  communication | .16 | .16 | .02 | .00 | .02 | -.08 | .06 | -.11 | -.17 | .06 | **.09** |

*Note*. Correlations are in the lower left triangle, variances are on the diagonal (bolded), and standardized residuals are in the upper right triangle.

**Supplementary Table S6A** Maternal Parenting Practice Domain Scores: International Samples

| Maternal Parenting Practice Domains | International  Sample  (*N* = 653) | | Argentina  (*n* = 139) | | Belgium  (*n* = 117) | | Brazil  (*n* = 40) | | France  (*n* = 59) | | Israel  (*n* = 31) | | | Italy  (*n* = 100) | | Japan  (*n* = 47) | | Kenya  (*n* = 30) | | U.S.  (*n* = 90) | |
| --- | --- | --- | --- | --- | --- | --- | --- | --- | --- | --- | --- | --- | --- | --- | --- | --- | --- | --- | --- | --- | --- |
|  | *M* | *SD* | *M* | *SD* | *M* | *SD* | *M* | *SD* | *M* | *SD* | *M* | *SD* | *M* | | *SD* | *M* | *SD* | *M* | *SD* | *M* | *SD* |
| Nurture | .00 | .61 | -.04 | .60 | -.11 | .63 | .10 | .47 | .15 | .69 | -.31 | .56 | .18 | | .56 | -.23 | .54 | .41 | .51 | -.04 | .60 |
| Physical | .10 | .11 | .11 | .10 | .05 | .06 | .09 | .09 | .08 | .08 | .05 | .06 | .05 | | .06 | .17 | .13 | .18 | .12 | .18 | .14 |
| Social | .00 | .71 | .36 | .97 | -.21 | .49 | .05 | .61 | .02 | .72 | -.18 | .56 | -.04 | | .62 | -.02 | .61 | -.32 | .63 | -.10 | .50 |
| Didactic | .00 | .92 | .26 | .95 | -.39 | .59 | -.09 | .80 | .17 | .96 | -.05 | .78 | .06 | | .94 | .20 | 1.17 | -.72 | .53 | .12 | 1.00 |
| Material | .00 | .66 | -.06 | .54 | .17 | .64 | -.36 | .45 | .26 | .59 | .63 | .76 | -.33 | | .59 | -.25 | .52 | -.70 | .49 | .38 | .61 |
| Language | .00 | .88 | .04 | .95 | .12 | .75 | -.05 | .71 | .15 | .80 | -.12 | .68 | .12 | | .86 | .04 | .83 | -1.35 | .61 | .05 | .89 |

**Supplementary Table S6B** Infant Behavior Domain Scores: International Samples

| Infant Behavior Domains | International  Sample  (*N* = 653) | | Argentina  (*n* = 139) | | Belgium  (*n* = 117) | | Brazil  (*n* = 40) | | France  (*n* = 59) | | Israel  (*n* = 31) | | | Italy  (*n* = 100) | | Japan  (*n* = 47) | | Kenya  (*n* = 30) | | U.S.  (*n* = 90) | |
| --- | --- | --- | --- | --- | --- | --- | --- | --- | --- | --- | --- | --- | --- | --- | --- | --- | --- | --- | --- | --- | --- |
|  | *M* | *SD* | *M* | *SD* | *M* | *SD* | *M* | *SD* | *M* | *SD* | *M* | *SD* | *M* | | *SD* | *M* | *SD* | *M* | *SD* | *M* | *SD* |
| Physical^a^ | 4.74 | .80 | 4.61 | .80 | 4.48 | .50 | 5.42 | .57 | 4.61 | .71 | 5.12 | .69 | 4.36 | | .54 | 4.80 | .64 | 5.20 | 1.14 | 5.20 | .95 |
| Social | .00 | .64 | .22 | .59 | .15 | .61 | -.08 | .57 | -.02 | .55 | .17 | .43 | -.33 | | .64 | -.44 | .55 | -.02 | .58 | .05 | .69 |
| Exploration | .00 | .58 | .00 | .56 | .00 | .57 | .06 | .41 | -.02 | .46 | .15 | .40 | -.27 | | .60 | .05 | .60 | -.26 | .63 | .32 | .57 |
| Nondistress vocalization | .00 | .92 | -.09 | .77 | .04 | .94 | -.32 | .71 | -.49 | 1.13 | .12 | .83 | .31 | | .92 | 0.01 | .84 | .60 | 1.00 | -.03 | .88 |
| Distress communication | .00 | .91 | .23 | 1.11 | -.08 | .94 | -.35 | .46 | -.26 | .61 | .26 | .93 | .00 | | .91 | .09 | .81 | .15 | 1.04 | -.11 | .74 |

^a^In months.

**Supplementary Table S7** Correlation Matrix, Variances, and Standardized Residuals for Final Model (Figure 2) for the International Sample

|  | 1 | 2 | 3 | 4 | 5 | 6 | 7 | 8 | 9 | 10 | 11 |
| --- | --- | --- | --- | --- | --- | --- | --- | --- | --- | --- | --- |
| 1. Mother | | | | | | | | | | | |
| 1 Nurture | **.37** | -.06 | -.02 | .02 | .04 | .04 | -.00 | -.12 | -.01 | .04 | .01 |
| 2 Physical | -.04 | **.03** | -.01 | .11 | .03 | -.11 | .01 | -.01 | .06 | .06 | .06 |
| 3 Social | .05 | .28 | **.02** | -.01 | -.01 | -.01 | -.00 | .07 | -.04 | .03 | .03 |
| 4 Didactic | -.20 | .24 | .41 | **.04** | .07 | .03 | -.06 | -.04 | -.01 | -.05 | .05 |
| 5 Material | -.31 | .03 | -.01 | .37 | **.44** | .04 | .03 | .05 | -.01 | -.05 | .01 |
| 6 Language | .11 | .03 | .47 | .42 | .13 | **.78** | -.03 | -.01 | .02 | -.04 | .00 |
| 1. Infant | | | | | | | | | | | |
| 7 Physical | -.14 | .25 | .03 | .05 | .17 | -.03 | **.00** | -.01 | .06 | .04 | -.03 |
| 8 Social | -.12 | .04 | .24 | .09 | .05 | .14 | -.01 | **.05** | .15 | .02 | -.02 |
| 9 Exploration | -.48 | .15 | -.03 | .38 | .48 | .02 | .27 | .15 | **.33** | -.08 | -.02 |
| 10 Nondistress  vocalization | -.06 | .06 | .03 | -.05 | -.05 | -.04 | .04 | .02 | -.08 | **.12** | .01 |
| 11 Distress  communication | .09 | .06 | .03 | .05 | .01 | .00 | -.03 | -.22 | -.15 | .25 | **.10** |

*Note*. Correlations are in the lower left triangle, variances are on the diagonal (bolded), and standardized residuals are in the upper right triangle.

**References**

1. Wilson EO. Sociobiology. Harvard University Press (1975).
2. Bjorklund DF, Myers AJ. The evolution of parenting and evolutionary approaches to childrearing. In: Bornstein MH, editors. Handbook of parenting.Vol. 2. Biology and ecology of parenting. 3rd ed. Routledge (2019). p. 3–29. doi: 10.4324/9780429401459-1
3. Geary DC. Evolution and proximate expression of human paternal investment. Psychol Bull. (2000) 126(1):55–77. doi: 10.1037/0033-2909.126.1.55
4. Moller AP. The evolution of monogamy: mating relationships, parental care, and sexual selection. In: Boesch C, editors. Monogamy: Mating strategies and partnerships in birds, humans, and other mammals. Cambridge University Press (2003). p. 29–41.
5. Holden GW, Miller PC. Enduring and different: a meta-analysis of the similarity in parents’ childrearing. *Psychol Bull.* (1999) 125:223-54. Doi: 10.1037/0033-2909.125.2.223
6. Leiderman PH, Tulkin SR, Rosenfeld A, editors. Culture and infancy: Variations in the human experience. Academic Press (1977).
7. Weisner TS, Gallimore R. My brother’s keeper: child and sibling caretaking. Curr Anthropol. (1977) 18:169–90. doi: 10.1086/201883
8. Parke RD, Cookston JT. Fathers and families. In: Bornstein MH, editors. Handbook of parenting. Vol. 3: Being and becoming a parent. 3rd ed. Routledge (2019). p. 64–136. doi: 10.4324/9780429433214-3
9. Bögels SM, Perotti EC. Does father know best? A formal model of the paternal influence on childhood social anxiety. J Child Fam Stud. (2011) 20(2):171–81. doi: 10.1007/s10826-010-9441-0
10. Konner M. The evolution of childhood: Relationships, emotions, mind. Belknap Press (2010).
11. Livingston G, Parker K. 8 Facts about American dads. Pew Research Center (2019). Available at: https://www.pewresearch.org/fact-tank/2019/06/12/fathers-dayfacts/
12. Negraia DV, Augustine JM, Prickett KC. Gender disparities in parenting time across activities, child ages, and educational groups. J Fam Issues. (2018) 39 (11):3006–28. doi: 10.1177/0192513x18770232
13. Fox J. Applied regression analysis, linear models and related methods. Sage (1997).
14. Tabachnick BG, Fidell LS. Using multivariate statistics. 7th ed. Pearson (2019).
15. Bollen KA. Outliers and improper solutions: a confirmatory factor analysis example. Sociol Methods Res. (1987) 15:375–84. doi: 10.1177/0049124187015004002
16. Bentler PM, Weeks DG. Linear structural equations with latent variables. Psychometrika. (1980) 45:289–308. doi: 10.1007/BF02293905
17. Bentler PM. EQS 6 Structural equations program manual. Multivariate Software (2008). Available at: www.mvsoft.com
18. Satorra A, Bentler PM. Corrections to test statistics and standard errors in covariance structure analysis. In: Von Eye A, Clogg CC, editors. Latent variable analysis: Applications for developmental research. Sage Publications (1994). p. 399–419.
19. Satorra A, Bentler PM. A scaled difference chi-square test statistic for moment structure analysis. Psychometrika. (2001) 66(4):507–14. doi: 10.1007/BF02296192
20. Bentler PM. Comparative fit indexes in structural models. Psychol Bull. (1990) 107(2):238–46. doi: 10.1037/0033-2909.107.2.238
21. Browne MW, Cudeck R. Alternative ways of assessing model fit. In: Bollen KA, Long JS, editors. Testing structural equation models. Sage (1993). p. 136–62.
22. Akaike H. Factor analysis and AIC. Psychometrika. (1987) 52:317–32. doi: 10.1007/BF02294359
23. Kaplan D. Structural equation modeling: Foundations and extension. Sage (2000).
24. Hu L-T, Bentler PM. Cutoff criteria for fit indexes in covariance structure analysis: conventional criteria versus new alternatives. Struct Equ Modeling. (1999) 6:1–55. doi: 10.1080/10705519909540118
25. Mardia KV. Measures of multivariate skewness and kurtosis with applications. Biometrika. (1970) 57:519–30. doi: 10.1093/biomet/57.3.519
26. Bentler, P. M. (1995). EQS structural equations program manual. Los Angeles, CA: BMDP Statistical Software, Inc.
27. Bollen KA. Structural equations with latent variables. John Wiley and Sons (1989). doi: 10.1002/9781118619179
28. Bornstein MH, Putnick DL, Esposito G. The nature and structure of mothers’ parenting their infants. *Parenting: Science and Practice*. (2022) 22(2):83–127. doi: 10.1080/15295192.2022.2057799

**Suggested Reading**

Ainsworth MDS, Blehar MC, Waters E, Wall S. Patterns of attachment: A psychological study of the strange situation. Erlbaum (1978).

Bowlby J. Attachment and loss: Vol. 1: Attachment. Hogarth (1969).

Byrne BM, Shavelson RJ, Muthén B. Testing for the equivalence of factor covariance and mean structures: the issue of partial measurement invariance. *Psychol Bull*. (1989) 105:456–66. doi: 10.1037/0033-2909.105.3.456

Crockenberg S, Litman C. Autonomy as competence in 2-year-olds: maternal correlates of child defiance, compliance, and self-assertion. *Dev Psychol*. (1990) 26(6):961–71. doi: 10.1037/0012-1649.26.6.961

Floyd FJ, Widaman KF. Factor analysis in the development and refinement of clinical assessment instruments. *Psychol Assess*. (1995) 7(3):286–99. doi: 10.1037/1040-3590.7.3.286

Gould SJ. Ontogeny and phylogeny. Harvard University Press (1977).

Hartmann DP. Assessing the dependability of observational data. *New Dir Methodol Soc Behav Sci*. (1982) 14:51–65. https://doi.org/10.1111/j.1600-0447.1982.tb00292.x

Hartmann DP. Considerations in the choice of interobserver reliability estimates. *J Appl Behav Anal*. (1977) 10(1):103–16. doi: 10.1901/jaba.1977.10-103

Hastings PD, Grusec JE. Parenting goals as organizers of responses to parentchild disagreement. *Dev Psychol*. (1998) 34(3):465–79. doi: 10.1037/0012-1649.34.3. 465

Hollenbeck AR. Problems of reliability in observational research. In: Sackett GP, editors. Observing behavior. Vol. 2. Data collection and analysis methods. University Park Press (1978). p. 79–98.

Jackson DN. Jackson personality inventory manual. Research Psychologists Press (1976).

Jöreskog KG, Sörbom D. LISREL VI: Analysis of linear structural relationships by the method of maximum likelihood. Scientific Software Inc. (1984).

MacCallum RC, Roznowski M, Necowitz LB. Model modifications in covariance structure analysis: the problem of capitalization on chance. *Psychol Bull*. (1992) 111:490–504. doi: 10.1037/0033-2909.111.3.490

Masten AS, Palmer AR. Parenting to promote resilience in children. In: Bornstein MH, editors. Handbook of parenting. Vol. 5. The practice of parenting. 3rd ed. Routledge (2019). p. 156–88. doi: 10.4324/9780429401695-6

Paunonen S, Jackson DN. The Jackson personality inventory and the five-factor model of personality. *J Res Pers*. (1996) 30:42–59. doi: 10.1006/jrpe.1996.0003

Penman R, Cross T, Milgram-Friedman J, Meares R. Mother’s speech to prelingual infants: a pragmatic analysis. *J Child Lang.* (1983) 10:17–34. doi: 10.1017/S0305000900005109

Pesch MH, Lumeng JC. Methodological considerations for observational coding of eating and feeding behaviors in children and their families. *Int J Behav Nutr Phys Act*. (2017) 14(1):1–14. doi: 10.1186/s12966-017-0619-3

Putnick DL, Bornstein MH. Measurement invariance conventions and reporting: the state of the art and future directions for psychological research. *Dev Rev*. (2016) 41:71–90. doi: 10.1016/j.dr.2016.06.004

Schaller M. The parental care motivational system and why it matters (for everyone). *Curr Dir Psychol Sci*. (2018) 27:295–301. doi: 10.1177/0963721418767873

Scott-Lennox JA, Lennox RD. Sex-race differences in social support and depression in older low-income adults. In: Hoyle RH, editors. Structural equation modeling: Concepts, issues, and applications. Sage Publications (1995) p. 199–216.

Tooby J, Cosmides L. The psychological foundations of culture. In: Barkow JH, Cosmides L, Tooby J, editors. The adapted mind: Evolutionary psychology and the generation of culture. Oxford University Press (1992). p. 19–136.

Trevarthen C, Aitken KJ. Infant intersubjectivity: research, theory, and clinical applications. *J Child Psychol Psychiatry*. (2001) 42:3–48. doi: 10.1111/1469-7610.00701

Trevarthen C, Hubley P. Secondary intersubjectivity: confidence, confiding and acts of meaning in the first year. In: Lock A, editors. Action gesture and symbol. Academic Press (1978). p. 183–229.

Worthman CM, DeCaro J, Brown R. Cultural consensus approaches to the study of American family life (MARIAL Working Paper 13). CCM and Working Families (2002). Available at: http://anthropology.emory.edu/home/documents/worthman-lab/Cultural%20consensus.pdf
